# Julio Óscar Trelles Montes (1904–1990)

**DOI:** 10.1007/s00415-026-14003-9

**Published:** 2026-07-15

**Authors:** João Pedro do Couto Caetano, Isadora Wiegand Mascarenhas, Alice Alves Petry

**Affiliations:** 1 Catholic University of Pelotas (UCPel), Rua Jaguarão, 1056, Laranjal, Pelotas, RS 96090-350 Brazil; 2 Postgraduate Program in Dentistry, Federal University of Pelotas (UFPel), Pelotas, RS Brazil

Julio Óscar Trelles Montes (Fig. 1) left two legacies: the first description of human neurolymphomatosis and a national school of neurology in Peru. He was born on 23 August 1904 in Andahuaylas, in the southern Peruvian Andes, and, after his schooling in Cusco and Lima, travelled to France to study medicine at the University of Paris, where he graduated in 1935 [1]. Admitted by competitive examination in 1929 to the internship of the Seine asylums, he was drawn into the orbit of the Dejerine Foundation and became an assistant and then head of the laboratory directed by Jean Lhermitte (1877–1959), where he absorbed the anatomo-clinical method of the French school; working almost always at his master’s side between 1930 and 1935, he produced the body of work that established his reputation, and in 1934 the Société Médico-Psychologique of Paris awarded him its Prix Trénel for his clinical work in psychiatry [1].

**Fig. 1:**
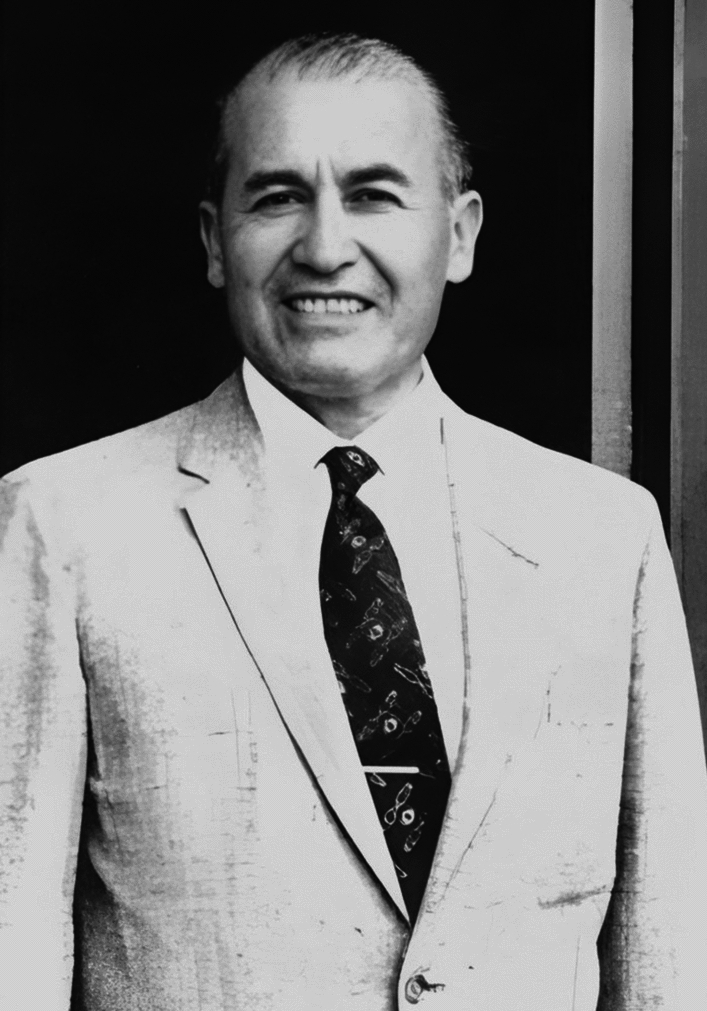
Julio Óscar Trelles Montes (1904–1990), pioneer Peruvian neurologist and statesman

His research belonged to the great French tradition of correlating focal lesions of the nervous system with their clinical expression, and it concentrated on the brainstem and posterior fossa. With Lhermitte he documented the hypertrophic degeneration of the inferior olivary nucleus [2]; and in 1935, reasoning that lesions of the inferior cerebellar peduncle never produced palatal myoclonus, he postulated a dentato-olivary pathway running from the dentate nucleus to the contralateral inferior olive by way of the superior cerebellar peduncle and the central tegmental tract [3]. The hypothesis was confirmed anatomically only decades later, and the circuit, now read as a limb of the dentato-rubro-olivary (Guillain–Mollaret) triangle, remains the accepted substrate of hypertrophic olivary degeneration and palatal tremor, today recognised on magnetic resonance imaging [3]. With Lhermitte and Lévy he furnished one of the early anatomo-clinical accounts of peduncular hallucinosis, tracing the vivid visual hallucinosis with preserved insight to a midbrain lesion confirmed at autopsy [4]. The achievement that anchored his name in the neurological lexicon also came in 1934, again with Lhermitte: the description of human peripheral neurolymphomatosis in a 67-year-old woman with a progressive malignant neuropathy whose peripheral nerves were infiltrated by lymphoid cells in the absence of systemic or cerebral disease, for which they proposed the term ‘peripheral lymphomatosis’ [5]. Now classified as a lymphoma of the peripheral nervous system (most often diffuse large B-cell in type, involving nerve roots, plexuses and cranial nerves, and long identifiable only at autopsy), neurolymphomatosis was once uniformly fatal but can today be recognised in life and treated with chemotherapy and immunotherapy, so that the entity he helped define remains clinically live and actively studied nearly a century on [6]. These brainstem studies culminated in his 1944 monograph *La oliva bulbar*, and the same anatomo-clinical spirit animated the textbook he coauthored with Pierre Masquin (1900–1976) under Lhermitte’s patronage, *Précis d’anatomo-physiologie normale et pathologique du système nerveux central* (Doin, Paris, 1937); a standard French reference, it reached a third edition revised by Julián de Ajuriaguerra (Doin, Paris, 1949) and a fourth in 1966.

More than a contributor to the French school, Trelles became the conduit through which its anatomo-clinical method reached South America [7]. He returned to Peru in 1935 [8] and revalidated his Paris degree at the Universidad Nacional Mayor de San Marcos with a thesis on pontine softening that was awarded the prize of the National Academy of Medicine [1]. He took over the old ‘El Refugio’ asylum for incurables in Lima and transformed it into the country’s first neurological hospital, Santo Toribio de Mogrovejo, which he directed from 1944 to 1974 and made the cradle of the neurosciences in Peru [8]. He held the chair of neurology at San Marcos until 1961, after which his teaching passed to the newly established Universidad Peruana Cayetano Heredia, of which he was a co-founder [8]; and, with the psychiatrist Honorio Delgado (1892–1969), he had launched the *Revista de Neuro-Psiquiatría*, whose first issue appeared in March 1938 and which carried European neuropathology to a Spanish-speaking readership [1]. Through hospital, chair and journal he trained the first generations of Peruvian neurologists and is regarded as the founder of the country’s neurological school: by the time he stepped down, neurology in Peru had its own hospital, its own journal and its own practitioners, where a generation earlier it had had none [8]. His research in Lima turned to the diseases of his own region: with Lazarte he produced an early systematic clinical, histopathological and parasitological study of cerebral cysticercosis, sorting its presentations into epileptic, hypertensive, psychic and apoplectic forms and giving order to a disorder that is still a leading cause of acquired epilepsy in the Andes, a subject to which he would return for the rest of his career [9]. His international standing was recognised by election to the Académie Nationale de Médecine and by honorary doctorates from Aix-en-Provence and the Sorbonne [1]. In 1963, the year he briefly served as Prime Minister of Peru, he presided over the inaugural Pan-American Congress of Neurology in Lima [7].

Trelles died in Lima on 2 October 1990, and in 1991 the National Institute of Neurological Sciences, the institutional successor of his hospital, was named after him. In his last years he had turned to chronicling his own discipline, writing biographical tributes to the masters who had shaped French and Peruvian neurology, among them his teacher Lhermitte [10], one of several such memoirs from his hand. Having recorded the lives of others, he is now himself remembered.

## Data Availability

Not applicable. All sources consulted are publicly available and cited in the reference list.
